# SIGNOR 2.0, the SIGnaling Network Open Resource 2.0: 2019 update

**DOI:** 10.1093/nar/gkz949

**Published:** 2019-10-29

**Authors:** Luana Licata, Prisca Lo Surdo, Marta Iannuccelli, Alessandro Palma, Elisa Micarelli, Livia Perfetto, Daniele Peluso, Alberto Calderone, Luisa Castagnoli, Gianni Cesareni

**Affiliations:** 1 Department of Biology, University of Rome Tor Vergata, Via della Ricerca Scientifica, 00133 Rome, Italy; 2 European Bioinformatics Institute (EMBL-EBI), European Molecular Biology Laboratory, Wellcome Genome Campus, Hinxton, Cambridgeshire CB10 1SD, UK; 3 IRCSS Fondazione Santa Lucia, 00142 Rome, Italy

## Abstract

The SIGnaling Network Open Resource 2.0 (SIGNOR 2.0) is a public repository that stores signaling information as binary causal relationships between biological entities. The captured information is represented graphically as a signed directed graph. Each signaling relationship is associated to an effect (up/down-regulation) and to the mechanism (e.g. binding, phosphorylation, transcriptional activation, etc.) causing the up/down-regulation of the target entity. Since its first release, SIGNOR has undergone a significant content increase and the number of annotated causal interactions have almost doubled. SIGNOR 2.0 now stores almost 23 000 manually-annotated causal relationships between proteins and other biologically relevant entities: chemicals, phenotypes, complexes, etc. We describe here significant changes in curation policy and a new confidence score, which is assigned to each interaction. We have also improved the compliance to the FAIR data principles by providing (i) SIGNOR stable identifiers, (ii) programmatic access through REST APIs, (iii) bioschemas and (iv) downloadable data in standard-compliant formats, such as PSI-MI CausalTAB and GMT. The data are freely accessible and downloadable at https://signor.uniroma2.it/.

## INTRODUCTION

Capturing, in a structured format, the intricate protein interaction web underlying cell signaling is a first step to comprehend how their deregulation can cause phenotype modulations and diseases (1). Signaling resources can be classified according to their representation model (2). Several resources collect molecular interaction data as undirected physical protein-protein interactions (MINT, IntAct, IMEx, BIOGRID) (3–5). Others (Reactome, KEGG-metabolism) have adopted the reaction-based model, where the relation is represented as a reaction where a catalyst modulates the transformation of an input entity into an output (6,7). Differently, databases such as SIGNOR (8), SignaLink (9) and KEGG-pathways (6) represent signaling data as activity flows. SIGNOR 2.0, the SIGnaling Network Open Resource 2.0 (https://signor.uniroma2.it/) stores manually annotated causal information extracted from the scientific literature as binary relationships between biological entities. In SIGNOR 2.0, each causal interaction is represented as a source entity that affects (up-regulates, down-regulates, etc.) a target entity and is annotated with the mechanism underlying the regulation (e.g. phosphorylation, ubiquitination, transcriptional regulation, etc.). Causal interactions are visualized as a dynamic and customizable graph where nodes are the entities and edges represent the causal relationships between them (10). Over the years SIGNOR 2.0 has established itself as the manually annotated database with highest coverage and annotation depth (2).

Here, we report the major improvements of the resource since its first release in 2016 (8). These include a significant growth in the number of causal interactions, in particular those involving mechanisms such as chemical inhibition/activation, phosphorylation, dephosphorylation and transcriptional regulation. Changes have been made in the curation practice and a manual of curation policy has been produced. In addition, we have revised the method to estimate a confidence score for each relationship. Finally, in order to comply with the FAIR data principles (11), new rules has been adopted such as (i) assignment of SIGNOR stable identifiers, (ii) programmatic access to data through REST APIs, (iii) bioschemas and downloadable data in standard-compliant formats such as PSI-MI CausalTAB (12) and GMT files. We have also considerably improved interoperability with other resources, such as UniProtKB (13), ChEBI (14), Complex Portal (15), PubMed, Gene Ontology (16,17) and BRENDA (18).

## RESULTS

### Data growth and statistics

**Figure 1.**
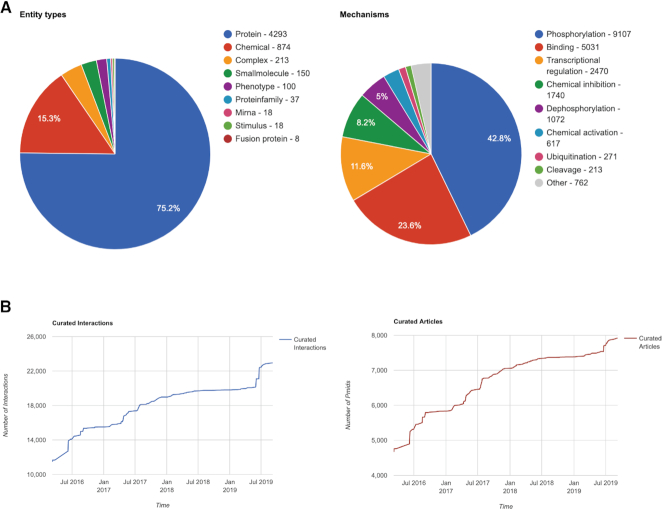
SIGNOR Data Growth. (**A**) The pie charts illustrate the number of entity types and mechanisms annotated in SIGNOR. (**B**) The growth curves illustrate the increment in the number of curated interactions and articles (B) since the 2016.

The present version of SIGNOR 2.0, September 2019, contains almost 23 000 causal interaction records (Figure 1) supported by 7921 publications. Thus, over the past three years, the number of interactions has almost doubled, and the number of curated papers has increased steadily (Figure 1). The coverage of the human proteome is still rather sparse as only 4294 proteins are annotated with a relationship that permits to link to the cell causal interactome. The graph that can be downloaded from SIGNOR 2.0 is a multipartite graph as its vertices, in addition to proteins, may also represent other entities such as chemicals (874), small molecules (150), complexes (213), phenotypes (100) and protein families. The pie charts in Figure 1A illustrate the relative abundance of the different entity types and annotated mechanisms, while Figure 1B details the growth of annotated interactions and curated articles over the years.

Most of the increment in the number of annotated interactions is from unbiased (domain-independent) curation. However, dedicated efforts were made to increase the coverage of domains of interest in our group. For instance, a specific literature search aimed at extending the coverage of relationships that modulate the activity of Transcription Factors (TFs) and the relationships between TFs and their validated target genes. To date, 571 TFs (35% of the TFs defined in the list of Lambert group) (19) have at least one relationship annotated in SIGNOR.

To support a group effort aimed at linking ‘cancer genes’ to cancer Hallmark phenotypes (20), we have also significantly increased the coverage of the cancer related genes that are annotated in SIGNOR 2.0: 497 of the 564 Tier 1 onco-related genes (88%) listed in the ‘Cancer Gene Census’ (21) are now integrated in the SIGNOR 2.0 causal network.

A dedicated curation effort was also devoted to post-translational modifications, focusing on phosphorylation and dephosphorylation modifications. Only modifications with a known effect (activation/inactivation) on the target protein were considered. As a consequence, the number of causal phosphorylation reactions have increased from 4900 to 9088 and the dephosphorylation reactions from 250 to 1072. 381 kinases and 119 phosphatases are now integrated in the SIGNOR causal network.

SIGNOR was also conceived as a resource to assist in the design of network perturbation experiments. Thus, a significant fraction of the recent data curation was devoted to increasing the number of relationships involving chemical ligands that affect the activity of target proteins. To this end, we downloaded the information annotated in the Guide to Immunopharmacology (www.guidetopharmacology.org) resource that contains expert-curated interactions between targets and ligands (drugs, chemicals, small molecules and proteins) extracted from pharmacology and drug discovery journals (22). In the curation of this dataset, we prioritized interactions between FDA-approved drugs, in particular anti-cancer targeted drugs, and as targets, proteins involved in important cellular processes and diseases such as receptors and TFs. We then mapped the ligands to their ChEBI or PubCHEM IDs and re-curated each paper following our curation rules and Controlled Vocabulary (CVs).

Among the curated ligand–target relationships, we have annotated 91 FDA-approved anti-cancer drugs and their 105 corresponding targets covering 60% of the anti-cancer drugs reported in the Sun paper (23). So far, this effort has allowed to annotate over 2350 chemical inhibition/activation relationships between over 1000 ligands (drugs, chemicals or small molecules) and 600 target proteins.

We have also annotated the large G protein-coupled receptors (GPCRs) interaction dataset recently published by Inoue *et al.* (24), covering ligand-induced interactions between 148 GPCRs and all 11 Gα subunits. This precious dataset is available in the download page (https://signor.uniroma2.it/downloads.php#GPCR).

SIGNOR 2.0 is mainly a non-organized collection of causal relationships. User feedback, however, has ascertained that the organization of the annotated relationships in pathways has some value for users. Since pathways are only useful mental abstractions, they cannot have a unique definition and different experts are likely to associate different relationships to the same pathway. Our criterion in defining the size of a subgraph representing a pathway is the ease in the interpretation of the corresponding graph layout. As a consequence, the pathways annotated in SIGNOR 2.0 are limited to a maximum of approximately 30 nodes and 50 edges. SIGNOR 2.0, in its latest release, offers the annotation of 49 curated pathways. This pathway list is available in the SIGNOR 2.0 home page through a drop-down menu that allows pathway selection for graphical representation. Moreover, the pathways stored in SIGNOR 2.0 can be viewed in the NDEx resource (25) and visualized and manipulated in the Cytoscape application (26).

### Curation policy

Over the past few years, the signaling community has worked within the HUPO-PSI and GREEKC frameworks (27), to develop guidelines, curation rules, controlled vocabularies and formats for the annotation and representation of causal interactions in a structured format. This effort aims at facilitating data exchange and comparison. The CausalTAB or ‘PSI Causal Interaction format TAB’, the newly developed PSI-MI tab-delimited format, has been established in agreement with the PSI-MI agenda (12,27) and new CV terms have been defined in order to be able to describe the diverse characteristics of causal interactions.

#### Minimum information and controlled vocabularies

In addition, the signaling community has agreed on establishing guidelines dubbed ‘minimum information about a molecular interaction causal statement (MI2CAST)’ that recommends ontologies, CVs and other related details to represent causal interactions in a standardized and reproducible way. We have updated our internal control vocabulary in compliance with the new standards. All terms have been mapped to the PSI-MI Controlled Vocabulary terms under the root term ‘causal interaction’ (https://www.ebi.ac.uk/ols/ontologies/mi/terms?iri=http%3A%2F%2Fpurl.obolibrary.org%2Fobo%2FMI_2233). For example, all the terms annotated as ‘activates/inactivates’ have been converted into ‘up-regulates/down-regulates’ or into more granular child terms (e.g. up-regulates activity or down-regulates quantity by destabilization).

#### Reference databases

All the biological entities represented in SIGNOR 2.0 are cross-linked to reference databases, such as UniProtKB (13) for proteins, miRbase (28) and RNA Central (29) for RNA, BRENDA (18) for cell lines/tissues. Following the MI2CAST recommendations, chemicals and small ligands are now linked to the ChEBI database (14) and, whenever the chemical is not annotated in this database, the reference database is PUBCHEM (30).

We have also annotated signaling interactions involving all the 18 antibodies utilized in cancer therapy and their cellular target (23) and four recombinant proteins currently used in cancer therapy. The antibody entities are linked to the reference database DRUGBANK (31) while the recombinant proteins refers to the ChEBI database (SID codes) (14).

SIGNOR 2.0 also contains data on fusion proteins, proteins generated by translocation events involving critical regions of proto-oncogenes. The rearrangements generate chimeric proteins directly promoting abnormal cell proliferation or differentiation blocks. Fusion proteins actively contributing to carcinogenesis, as for example the fusion proteins AML1-ETO and PML-RARα in Leukemia (32–34). Fusion proteins are very often annotated in UniProtKB (13) as unreviewed and with a very low annotation score. For this reason, we have decided to assign to these proteins an internal unique SIGNOR identifier (https://signor.uniroma2.it/relation_result.php?id=SIGNOR-FP1).

### Additional new features

#### Orthology mapping

SIGNOR 2.0 data curation maps all the experimental information to the human proteome while maintaining the information on the organism used to provide the experimental evidence. To meet some user requirements, an effort has been made to map the human causal interactome onto the proteome of two experimental organisms, *Mus musculus* and *Rattus norvegicus*. Mapping was obtained by extracting orthology data from the InParanoid resource (35). Each human protein was mapped to the mouse (or rat) protein with the highest orthology score. A script was also created in order to map SIGNOR 2.0 phosphorylation data onto the ortholog proteins using a BLOSUM75 scoring similarity matrix to align phosphopeptides in orthologs of different organisms. Thus, SIGNOR 2.0 is the union of three organism specific databases and the user can select in the home page the proteome context he/she wants to perform the searches in.

#### Improved search box

To provide a more intuitive user experience, the newly improved search box in the home page includes now the possibility to search by PMID by selecting the corresponding tab. The search result will include information about the selected article and any relations whose curations were based on it.

#### Shortest path

To meet user requests, we also implemented a new functionality that permits to connect any two graph nodes (entities) by causal interactions. In the search box, the user has now the possibility to insert one, two or more entity names or identifiers and, depending on the number of inserted entities, to get access to different functionalities. If only one entity is entered in the field, the search returns the ‘entity page’ listing all the relationships pertaining to the query entity. If the entity names are more than one, by clicking the ‘all’ check-box button, one obtains a graph showing all the relationships in which the query entities are involved, when the ‘connect’ check-box is selected only the interactions connecting the query entities will be displayed. An extension of this latter functionality permits to draw graphs connecting the query proteins via additional ‘bridge’ proteins (‘include first neighbors’ check-box).

**Figure 2.**
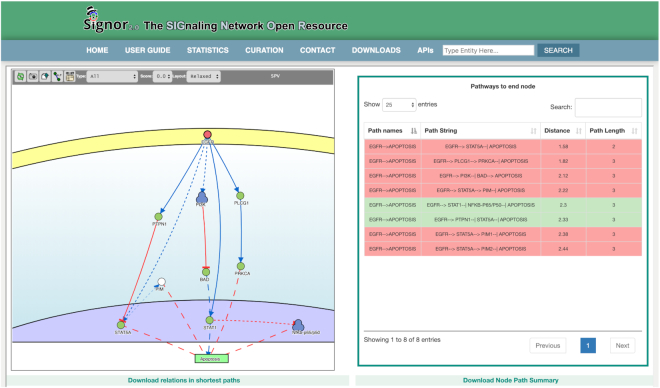
SIGNOR ‘shortest path’ functionality. In the homepage, the search panel allows to enter two SIGNOR entities such as genes (Uniprot ID or gene names) or phenotypes and to query the database for the shortest causal path(s) joining them. The result network displays the shortest paths linking the query entities and a table listing all the shortest paths. In the table, paths are colored in green or red depending on their activation or and inhibition output.

Finally, a new algorithm was implemented to identify the shortest directional path between any two nodes in the causal graph. The algorithm can be launched when the query field contains two entities. In this case the ‘shortest path’ check box can be selected to launch an algorithm that finds the shortest directional path (and those that are one step longer) between the two entities. The results of such a query are shown both in a graph and a table view (Figure 2), where the paths are ordered according to ‘path length’. The table view also shows, with a color-code whether each path results in activation or inhibition of the end entity (Figure 2).

#### Download page

The data in SIGNOR 2.0 can be freely downloaded. In the download page, the user can choose to download various subsets of the data, i.e phosphorylation data, specific interactions based on relation ID, transcriptional relations and pathway-specific relations in various formats, such as SBML and GMT. The entire dataset of binary causal interactions is offered both as a stable quarterly release and as a ‘live’, up-to-date file containing all the recently curate interactions. Files are offered in a tab-delimited format as well as a CausalTab. SIGNOR 2.0 entity data, and detailed information about CV, is available for download as well.

#### APIs

To facilitate programmatic access to SIGNOR 2.0, data can also be retrieved via a REST API that is based on simple URL queries using selected parameters, as shown in Supplementary Table S1. The query results are provided in a tab-delimited, plain text format.

#### Bioschemas

In order to join the effort toward increasing ‘searchability’ and ‘findability’ of biological data, the Schema.org markup has been included in the main SIGNOR 2.0 page code. This includes descriptive information about the database and its contents, plus information about data formats and retrieval.

### New SIGNOR 2.0 score

The new SIGNOR 2.0 score combines four features, each taken as evidence of the support for the functional relevance of the considered causal relationship. The features can be divided into two categories; the first one is based on the annotation within SIGNOR 2.0, whereas the second one employs external resources.

For the first two features, we count (i) the number of annotated articles (PMIDs) reporting works that support the interaction in the annotation in SIGNOR 2.0 and (ii) the number of occurrences of the specific relationship in a pathway that is already annotated in SIGNOR 2.0.

The remaining two features are independent from SIGNOR 2.0 annotations and consider the support that is possible to extract from two of the major curated pathway databases: Reactome (7) and UniProtKB (13).

For the ‘Reactome feature’ we count the number of occurrences of the specific interaction pair in the ‘Human Protein Interactions’ file that can be downloaded from the Reactome website. As for the UniProtKB feature, we surmise that if entity X (protein or phenotype) is mentioned in the UniprotKB page of protein Y, the two entities are more likely to be functionally correlated. Thus, for a given *source*–*target* relationship we count how many times the *target* entity is mentioned in the UniProtKB entity page of the *source*.

Each feature returns a figure ‘*n*’ (number of PMIDs, number of occurrences, number of mentions, etc.) that we associate to a ‘partial score’ *Y* according to the following formula in order to normalize each value in the 0–1 range.

$$\begin{equation*}{Y_f}\ = \ 1 - {C^{ - {n_f}}}\end{equation*}$$

The constant *C* was empirically set to 1.5 for all the features with the exception of the number of supporting PMIDs that was set to 2.2, since this feature has much lower values than the other ones. The final combined score (*r*) is obtained by averaging the four partial scores.

$$\begin{equation*}r\ = \frac{1}{4}\ \ \mathop \sum \limits_{i\ = \ 1}^4 {Y_{{f_i}}}\end{equation*}$$

The score ranges between 0 and 1. However the score can never be 0 since all SIGNOR relations are supported by at least one reference. The combined score distribution is depicted as a bar diagram (Supplementary Figure S1).

## DISCUSSION

Signaling information can be captured by a variety of different models each with a different level of description granularity and coverage potential. Some established databases, such as KEGG and REACTOME use reaction-based models, which capture some mechanistic details and—kinetic constants available—the dynamics of networks containing up to a few dozen entities. Models based on networks of logic relationships, such as SIGNOR, on the other hand, have a higher potential to describe larger systems at the expenses of mechanistic details. Users have the possibility to choose a specific resource in the signaling resources panorama, according to their needs or interest.

Here we have presented SIGNOR 2.0, which is a much improved version of the database published in 2016 (8). The most significant progresses are:

- The number of stored logic relationships has almost doubled.
- Focused curation on relationships involving membrane receptors, proteins mutated in cancers and pharmacologically relevant proteins.
- Clearer definition of curation rules, controlled vocabularies and standards for data exchange.
- REST APIs for programmatic access.
- New graph analysis tools to explore functional connections between any pair of network entities.

**Figure 3.**
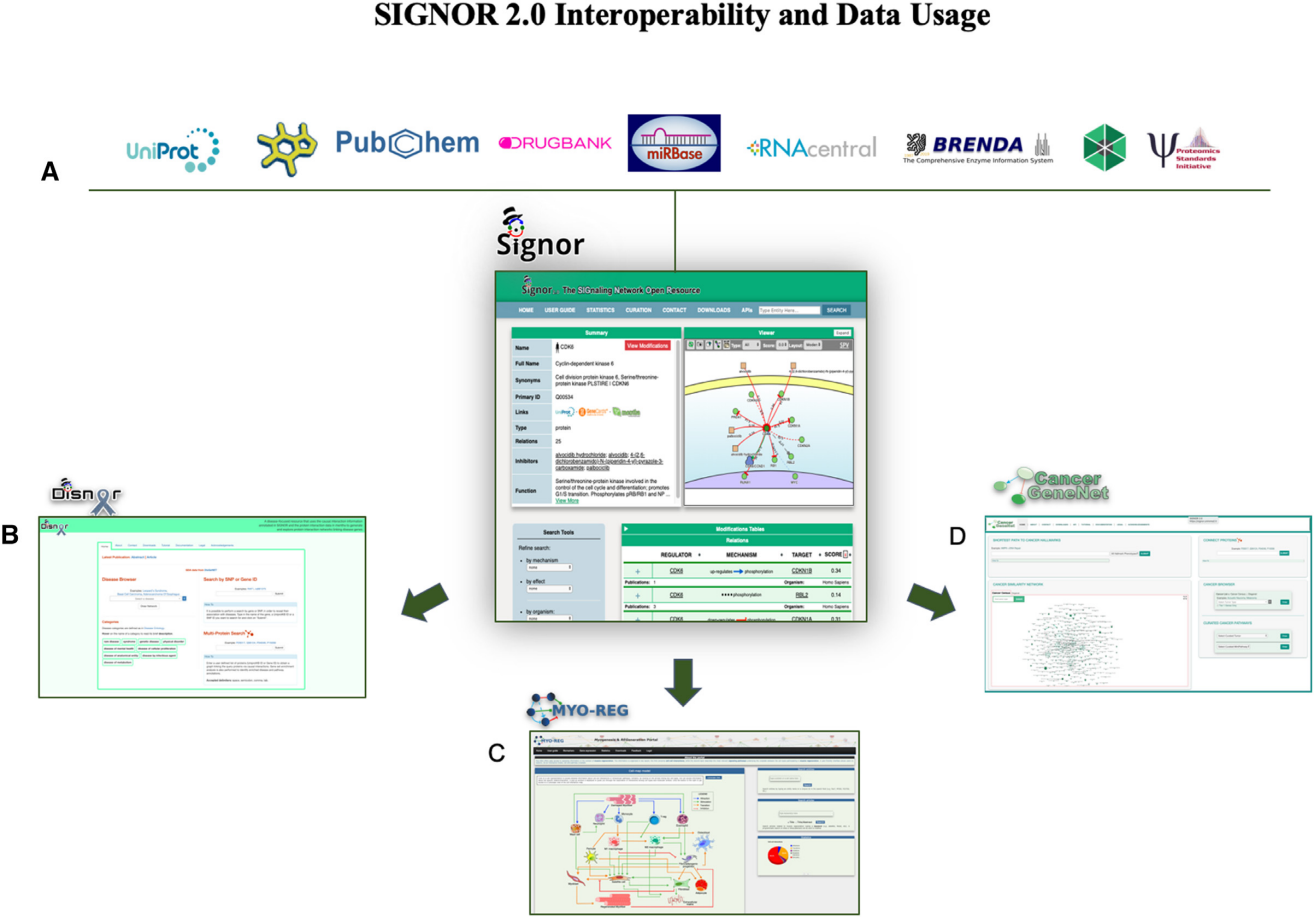
SIGNOR 2.0 interoperability and data usage. (**A**) SIGNOR 2.0 refers to a number of external resources for cross reference and metadata. The three specialized resources illustrated here use the data in SIGNOR to extract biologically relevant information in three biological domains: (**B**) Disnor, a resource designed to infer disease pathways by functionally linking disease-associated genes, (**C**) Myo-REG, a web portal that offers information on cell and signaling interactions during muscle regeneration and (**D**) CancerGeneNet, a resource that links genes cancer frequently mutated in cancer to cancer Hallmarks phenotypes.

The large collection of manually-curated logic relationships annotated in SIGNOR 2.0 can be used to infer the cell information flow and to reveal how mutations may cause diseases. Graph algorithms help to extract biological hypothesis from the stored information and dedicated resources build on the SIGNOR 2.0 dataset to offer biological insights in specific biological domains, such as diseases (36), cancer (Iannuccelli *et al.* under submission to the NAR database issue) and muscle regeneration (37) (Figure 3). However, users should bear in mind that these tools only offer ‘naive’ hypotheses based on graph theory, which should be critically evaluated.

The annotated causal relationships are supported by experimental evidence in different organisms and cell systems. Although SIGNOR 2.0 keeps track of this information, the relationships are automatically mapped onto the human proteome and then remapped by orthology, onto the proteome of other model organisms. The result is an intricate highly connected network. The impact of altering the activity of a specific node (mutation) on any other node of the network can be estimated by looking the graph paths connecting the two query nodes. The result of such a procedure is not unambiguous as the different paths that are retrieved may predict opposite effects on the target protein or phenotype. There are a variety of reasons for this apparently contradictory response. For instance, not all proteins are equally expressed in the different systems and a different proteome context may favor one or the other outcome. Although the newly developed reliability score may help in limiting the analysis to paths covering more trustworthy relationships, it is left to the ‘expert’ users to filter out the paths that, based on their experience, are unlikely to be biologically relevant or to test them experimentally.

SIGNOR 2.0, with its growth and improvements, is now a more mature resource that offers valuable tools to design experiments and interpret the results.

Importantly, in the new version of SIGNOR 2.0, we followed FAIR data principles in order to increase data interoperability. SIGNOR 2.0 now adopts stable identifiers, offers REST APIs for programmatic access, implements bioschemas and allows data download in standard-compliant format.

## DATA AVAILABILITY

SIGNOR 2.0 interaction data are available and freely downloadable at: https://signor.uniroma2.it/downloads.php.

Furthermore, programmatic access is available through the APIs section at https://signor.uniroma2.it/APIs.php.

All described database features are accessible via the main SIGNOR 2.0 website: https://signor.uniroma2.it/.

## Supplementary Material

gkz949_Supplemental_File

## References

[1] Barabási A.-L., Oltvai Z.N. Network biology: understanding the cell's functional organization. Nat. Rev. Genet. 2004; 5:101–113. 14735121 10.1038/nrg1272

[2] Türei D., Korcsmáros T., Saez-Rodriguez J. OmniPath: Guidelines and gateway for literature-curated signaling pathway resources. Nat. Methods. 2016; 13:966–967. 27898060 10.1038/nmeth.4077

[3] Oughtred R., Stark C., Breitkreutz B.J., Rust J., Boucher L., Chang C., Kolas N., O’Donnell L., Leung G., McAdam R. et al. The BioGRID interaction database: 2019 update. Nucleic Acids Res. 2019; 47:D529–D541. 30476227 10.1093/nar/gky1079 PMC6324058

[4] Licata L., Orchard S The MIntAct Project and Molecular Interaction Databases. Data Mining Techniques for the Life Sciences. 2016; 1415:NY Springer 55–69. 10.1007/978-1-4939-3572-7_3 27115627

[5] Orchard S., Kerrien S., Abbani S., Aranda B., Bhate J., Bidwell S., Bridge A., Briganti L., Brinkman F.S.L., Cesareni G. et al. Protein interaction data curation -The International Molecular Exchange Consortium (IMEx). Nat. Methods. 2012; 9:345–350. 22453911 10.1038/nmeth.1931 PMC3703241

[6] Kanehisa M., Furumichi M., Tanabe M., Sato Y., Morishima K. KEGG: New perspectives on genomes, pathways, diseases and drugs. Nucleic Acids Res. 2017; 45:D353–D361. 27899662 10.1093/nar/gkw1092 PMC5210567

[7] Sidiropoulos K., Viteri G., Sevilla C., Jupe S., Webber M., Orlic-Milacic M., Jassal B., May B., Shamovsky V., Duenas C. et al. Reactome enhanced pathway visualization. Bioinformatics. 2017; 33:3461–3467. 29077811 10.1093/bioinformatics/btx441 PMC5860170

[8] Perfetto L., Briganti L., Calderone A., Perpetuini A.C., Iannuccelli M., Langone F., Licata L., Marinkovic M., Mattioni A., Pavlidou T. et al. SIGNOR: A database of causal relationships between biological entities. Nucleic Acids Res. 2016; 44:D548–D554. 26467481 10.1093/nar/gkv1048 PMC4702784

[9] Fazekas D., Koltai M., Türei D., Módos D., Pálfy M., Dúl Z., Zsákai L., Szalay-Bek M., Lenti K., Farkas I.J. et al. SignaLink 2 – a signaling pathway resource with multi-layered regulatory networks. BMC Syst. Biol. 2013; 7:7. 23331499 10.1186/1752-0509-7-7 PMC3599410

[10] Calderone A., Cesareni G. SPV: a JavaScript Signaling Pathway Visualizer. Bioinformatics. 2018; 34:2684–2686. 29590303 10.1093/bioinformatics/bty188 PMC6061680

[11] Wilkinson M.D., Dumontier M., Aalbersberg I.J., Appleton G., Axton M., Baak A., Blomberg N., Boiten J.-W., da Silva Santos L.B., Bourne P.E. et al. The FAIR Guiding Principles for scientific data management and stewardship. Scientific Data. 2016; 3:160018. 26978244 10.1038/sdata.2016.18 PMC4792175

[12] Perfetto L., Acencio M.L., Bradley G., Cesareni G., Del Toro N., Fazekas D., Hermjakob H., Korcsmaros T., Kuiper M., Lægreid A. et al. CausalTAB: the PSI-MITAB 2.8 updated format for signalling data representation and dissemination. Bioinformatics. 2019; 35:3779–3785. 30793173 10.1093/bioinformatics/btz132 PMC6896241

[13] UniProt Consortium UniProt: a worldwide hub of protein knowledge. Nucleic Acids Res. 2019; 47:D506–D515. 30395287 10.1093/nar/gky1049 PMC6323992

[14] Hastings J., Owen G., Dekker A., Ennis M., Kale N., Muthukrishnan V., Turner S., Swainston N., Mendes P., Steinbeck C. ChEBI in 2016: improved services and an expanding collection of metabolites. Nucleic Acids Res. 2016; 44:D1214–D1219. 26467479 10.1093/nar/gkv1031 PMC4702775

[15] Meldal BH, Forner-Martinez O, Costanzo MC, Dana J, Demeter J, Dumousseau M, Dwight SS, Gaulton A, Licata L, Melidoni AN et al. The complex portal–an encyclopaedia of macromolecular complexes. Nucleic Acids Res. 2014; 43:D479–D478. 25313161 10.1093/nar/gku975 PMC4384031

[16] Ashburner M., Ball C.A., Blake J.A., Botstein D., Butler H., Cherry J.M., Davis A.P., Dolinski K., Dwight S.S., Eppig J.T. et al. Gene ontology: tool for the unification of biology. The Gene Ontology Consortium. Nat. Genet. 2000; 25:25–29. 10802651 10.1038/75556 PMC3037419

[17] Gene Ontology Consortium Gene Ontology Consortium: going forward. Nucleic Acids Res. 2015; 43:D1049–D1056. 25428369 10.1093/nar/gku1179 PMC4383973

[18] Jeske L., Placzek S., Schomburg I., Chang A., Schomburg D. BRENDA in 2019: a European ELIXIR core data resource. Nucleic Acids Res. 2019; 47:D542–D549. 30395242 10.1093/nar/gky1048 PMC6323942

[19] Lambert S.A., Jolma A., Campitelli L.F., Das P.K., Yin Y., Albu M., Chen X., Taipale J., Hughes T.R., Weirauch M.T. The Human Transcription Factors. Cell. 2018; 175:598–599. 30290144 10.1016/j.cell.2018.09.045

[20] Hanahan D., Weinberg R.A. Hallmarks of cancer: the next generation. Cell. 2011; 144:646–674. 21376230 10.1016/j.cell.2011.02.013

[21] Sondka Z., Bamford S., Cole C.G., Ward S.A., Dunham I., Forbes S.A. The COSMIC Cancer Gene Census: describing genetic dysfunction across all human cancers. Nat. Rev. Cancer. 2018; 18:696–705. 30293088 10.1038/s41568-018-0060-1 PMC6450507

[22] Harding S.D., Sharman J.L., Faccenda E., Southan C., Pawson A.J., Ireland S., Gray A.J.G., Bruce L., Alexander S.P.H., Anderton S. et al. The IUPHAR/BPS Guide to pharmacology in 2018: updates and expansion to encompass the new guide to immunopharmacology. Nucleic Acids Res. 2018; 46:D1091–D1106. 29149325 10.1093/nar/gkx1121 PMC5753190

[23] Sun J., Wei Q., Zhou Y., Wang J., Liu Q., Xu H. A systematic analysis of FDA-approved anticancer drugs. BMC Syst. Biol. 2017; 11:87. 28984210 10.1186/s12918-017-0464-7 PMC5629554

[24] Inoue A., Raimondi F., Kadji F.M.N., Singh G., Kishi T., Uwamizu A., Ono Y., Shinjo Y., Ishida S., Arang N. et al. Illuminating G-Protein-Coupling Selectivity of GPCRs. Cell. 2019; 177:1933–1947. 31160049 10.1016/j.cell.2019.04.044 PMC6773469

[25] Pillich R.T., Chen J., Rynkov V., Welker D., Pratt D. NDEx: a community resource for sharing and publishing of biological networks. Methods Mol. Biol. 2017; 1558:271–301. 28150243 10.1007/978-1-4939-6783-4_13

[26] Shannon P., Markiel A., Ozier O., Baliga N.S., Wang J.T., Ramage D., Amin N., Schwikowski B., Ideker T. Cytoscape: a software environment for integrated models of biomolecular interaction networks. Genome Res. 2003; 13:2498–2504. 14597658 10.1101/gr.1239303 PMC403769

[27] Hermjakob H., Montecchi-Palazzi L., Bader G., Wojcik J., Salwinski L., Ceol A., Moore S., Orchard S., Sarkans U., von Mering C. et al. The HUPO PSI’s molecular interaction format–a community standard for the representation of protein interaction data. Nat. Biotechnol. 2004; 22:177–183. 14755292 10.1038/nbt926

[28] Kozomara A., Birgaoanu M., Griffiths-Jones S. miRBase: from microRNA sequences to function. Nucleic Acids Res. 2019; 47:D155–D162. 30423142 10.1093/nar/gky1141 PMC6323917

[29] The RNAcentral Consortium RNAcentral: a hub of information for non-coding RNA sequences. Nucleic Acids Res. 2019; 47:D221–D229. 30395267 10.1093/nar/gky1034 PMC6324050

[30] Kim S., Chen J., Cheng T., Gindulyte A., He J., He S., Li Q., Shoemaker B.A., Thiessen P.A., Yu B. et al. PubChem 2019 update: improved access to chemical data. Nucleic Acids Res. 2019; 47:D1102–D1109. 30371825 10.1093/nar/gky1033 PMC6324075

[31] Wishart D.S., Feunang Y.D., Guo A.C., Lo E.J., Marcu A., Grant J.R., Sajed T., Johnson D., Li C., Sayeeda Z. et al. DrugBank 5.0: a major update to the DrugBank database for 2018. Nucleic Acids Res. 2018; 46:D1074–D1082. 29126136 10.1093/nar/gkx1037 PMC5753335

[32] De Braekeleer E., Douet-Guilbert N., De Braekeleer M. RARA fusion genes in acute promyelocytic leukemia: a review. Expert Rev Hematol. 2014; 7:347–357. 24720386 10.1586/17474086.2014.903794

[33] Martens J.H.A., Stunnenberg H.G. The molecular signature of oncofusion proteins in acute myeloid leukemia. FEBS Lett. 2010; 584:2662–2669. 20388510 10.1016/j.febslet.2010.04.002

[34] Licht J.D. AML1 and the AML1-ETO fusion protein in the pathogenesis of t(8;21) AML. Oncogene. 2001; 20:5660–5679. 11607817 10.1038/sj.onc.1204593

[35] Sonnhammer E.L.L., Östlund G. InParanoid 8: orthology analysis between 273 proteomes, mostly eukaryotic. Nucleic Acids Res. 2015; 43:D234–D239. 25429972 10.1093/nar/gku1203 PMC4383983

[36] Lo Surdo P., Calderone A., Iannuccelli M., Licata L., Peluso D., Castagnoli L., Cesareni G., Perfetto L. DISNOR: A disease network open resource. Nucleic Acids Res. 2018; 46:D527–D534. 29036667 10.1093/nar/gkx876 PMC5753342

[37] Palma A., Perpetuini A.C., Ferrentino F., Fuoco C., Gargioli C., Giuliani G., Iannuccelli M., Licata L., Micarelli E., Paoluzi S. et al. Myo-REG: A Portal for Signaling Interactions in Muscle Regeneration. Front Physiol. 2019; 10:1216. 31611808 10.3389/fphys.2019.01216 PMC6776608

