# Supplementary material for: SIGNOR 2.0, the SIGnaling Network Open Resource 2.0: 2019 update
Source: Nucleic Acids Res. 2019 Oct 29;48(D1):D504–10. doi: 10.1093/nar/gkz949 (PMC7145695; doi:10.1093/nar/gkz949)
Supplement: gkz949_Supplemental_File [file gkz949_supplemental_file.pdf]

Supplementary file 1

Methods

SIGNOR 2.0 Data Curation

The first step of the curation process consists in the systematic search of articles containing signaling information about the entities (protein, RNA, chemical, etc), the effect (up/down-regulation) and the mechanisms (phosphorylation, binding, chemical activation, etc) involved in the signaling interaction. Published literature is searched either manually or semi-automatically using text mining tools to identify papers that potentially describe causal interactions. Each selected paper is then evaluated and annotated by our trained curator staff.
During the curation process, curators use a tab delimited text file organized in three parts (entity, relationship and reference), every part contains mandatory fields to be filled with fundamental information that describe the logic relation. In order to ensure data interoperability and reproducibility, a new controlled vocabulary (CV) has been developed in order to represent the direction and sign of causal interactions in a structured format (1). Once data are entered in the database they can be downloaded in the PSI-MI tab-delimited format, CausalTAB (https://signor.uniroma2.it/downloads.php)

New causal terms added to PSI-MI CV:

Causal interactor type (entity types). The new 'Causal interactor type' term has been added in order to define new 'interactor types', such as 'stimulus' and 'phenotype'.

Biological Role (directionality). Two new 'biological role' parent terms: 'regulator' (MI:2274) and 'regulator target' (MI:2275) has been defined.

Interaction Type (mechanism). The 'Interaction type' column contains terms to describe the type of relationship between entities involved in a causal interaction, called 'mechanism' in SIGNOR 2.0. Terms such as 'phosphorylation reaction' or 'physical association' or 'ubiquitination reaction' were already present in the MI-CV, under the 'Interaction type' CV terms. The 'Causal Regulatory Mechanism' contains the new terms 'post transcriptional regulation', 'transcriptional regulation' and 'translation regulation', where the effect of entity A is not necessary immediately upstream the entity B. When the effect of entity A is not immediately upstream the entity B, these terms are associated with the term 'Functional Association' at the 'Interaction Type' level. The full set of terms is available at https://www.ebi.ac.uk/ols/ontologies/mi/terms?iri=http%3A%2F%2Fpurl.obolibrary.org%2Fobo%2FMI_2233.

Each interaction curated in SIGNOR 2.0 must report information on the following fields:

ENTITIES
•Molecule Type (*e.g.* chemical, proteins, protein family, complex, miRNA, phenotype);
•Molecule identifiers (*e.g.*UniprotKB id, ChEBI, Signor ID);

RELATIONSHIP
•Effect (up/down- regulates);
•Mechanism (*e.g.* binding, phosphorylation, transcriptional activation);
•Modified residue (*i.e.* Ser36)

•Organism, cell line, tissue
•Direct (yes/no)

REFERENCE
•PMID
•Short sentence

ENTITIES:
Type
In the Entities part curators annotate information about the Molecule Type of the source and the target entities. Molecule Type in SIGNOR 2.0 belong to ten different categories:

Protein
Complex
miRNA
Small Molecule
Protein family
Fusion protein
Chemical
Phenotype
Stimulus
Antibody

Identifiers
Each molecular entity is associated with a unique identifier and linked to a reference database.

Protein: UniProtKB
Complex: SIGNOR ID
miRNA: mirBase and RNA Central
Small Molecule: ChEBI and PUBCHEM
Protein family: SIGNOR ID
Fusion protein: SIGNOR ID
Chemical: ChEBI and PUBCHEM
Phenotype: SIGNOR ID
Stimulus: SIGNOR ID
Antibody: DRUGBANK

Directionality
In Signor 2.0, each interaction has a directionality, with a regulatory entity (Entity A) that acts on a regulated entity (Entity B).

RELATIONSHIP:

Effect
In the effect part, curators provide information about the effect (positive or negative) that the regulatory entity has on a regulated entity. A regulatory entity can act by up-regulating or down-regulating the function of another entity. It is also possible to specify if the regulation acts on the activity or the

quantity of the entity modulated through the regulation of expression or stability (e.g. down-regulates quantity by expression or up-regulates quantity by stabilization).

Mechanism
In the mechanism part, curators add details about the molecular mechanism of the interaction (e.g. phosphorylation, transcriptional regulation, etc.). Depending on the type of mechanism, the interaction type associated to the interaction can be direct or indirect.

Modified residue
If in the supporting manuscript, there are experimentally evidence that show which is the amino acid residue that undergoes the PTM, this information is also annotated.

Organism, cell lines/tissues
Information about the organism, cell line and tissue in which the interaction has been experimentally observed is included and cross referenced to the appropriate NCBI taxID, with the addition of the BRENDA IDs (2) for cell lines/tissues or -1 to indicate 'in vitro' experiments.

Direct
In the ‘direct’ field, curators annotate if relations between entities is direct or indirect.
Our curation rules state that the relations between entities involving binding or any Post-Translational Modification (PTM) is always considered direct, while interactions involving an entity and a phenotype is always annotated as indirect (3). Some mechanisms, such as transcriptional regulation, can have associated either the interaction type ‘direct or indirect’ depending on the reported information. We consider ‘direct’ those transcription relationships in which it is clear that the regulator (transcription factor) binds the promoter of the target gene by activating it (e.g. P53 binds to the MDM2 promoter and activates MDM2), while ‘indirect’ when there are several steps between the two entities involved in the interaction (e.g. EGFR activates FOS).

REFERENCE:
In the reference part, curators annotate the PubMed ID (PMID) of the supporting manuscript and a sentence, extracted from the same manuscript, possibly containing all the information captured in the entry. All publications have a PMID cross-reference associated.

SIGNOR, new technical features.

SIGNOR’s database is managed through PostgreSQL (v.8.4) and its data is accessed and manipulated using PHP. The web interface was also built using PHP, as well as HTML (HyperText Markup Language) and JavaScript.

Interoperability

As described above, a substantial effort has been made in order to support interoperability between resources. This has involved the annotation of entities using external cross-references from widely used databases (e.g. UniprotKB (4), BRENDA (2), etc.) for crosslinking purposes within the websites and to increase the findability of related information on the Web. Even in the instance of internally created entities, an effort has been made to match them, whenever possible, to external resources (e.g. ComplexPortal (5)).

Reliability score methods

We programmatically accessed Reactome (6) relations using the data available in the download section (https://reactome.org/download/current/interactors/reactome.homo_sapiens.interactions.tab-delimited.txt) in the same way we accessed the SIGNOR relation and pathway data. As for the UniProtKB (4) feature we gained the data using the *bioservices.uniprot* module. Finally, all the features were parsed and combined using *R* language.

Shortest path functionality

The shortest path functionality was implemented using ”*all_shortest_path”* function of the R *igraph* package (7) . In addition to explore the paths that are one step longer than the shortest one, we exploited another function (*all_simple_paths)* available in the Python *NetworkX* module (8). The result page shows the retrieved paths in a graph display and a summary table that provides two additional information associated to each path:

(i) the "*Distance*" that is related to path reliability. This metrics is computed as the sum of each step distance (d), where the distance is obtained by 1- r, where r is the new reliability score associated to each relation, as described in the paragraph (new SIGNOR 2.0 score). Path "*Distance*" was obtaine by the following formula, where N is the number of relation-steps in a path.

$$D_{path} = \sum_{rel=1}^{N} (1 - r_{rel})$$

(ii) Since each relationship has a sign (activates, inactivates) i t is possible to evaluate whether the path results in activation or inactivation of the target entity depending on the odd or even number of inhibitory steps. This is reported with a color background for each path: positive (green), negative (red) or unknown (gray).

References

1. Perfetto,L., Acencio,M.L., Bradley,G., Cesareni,G., Del Toro,N., Fazekas,D., Hermjakob,H., Korcsmaros,T., Kuiper,M., Lægreid,A., *et al.* (2019) CausalTAB: the PSI-MITAB 2.8 updated format for signalling data representation and dissemination. *Bioinformatics*, 10.1093/bioinformatics/btz132.

2. Jeske,L., Placzek,S., Schomburg,I., Chang,A. and Schomburg,D. (2019) BRENDA in 2019: a European ELIXIR core data resource. *Nucleic Acids Res.*, **47**, D542–D549.

3. Perfetto,L., Briganti,L., Calderone,A., Perpetuini,A.C., Iannuccelli,M., Langone,F., Licata,L., Marinkovic,M., Mattioni,A., Pavlidou,T., *et al.* (2016) SIGNOR: A database of causal relationships between biological entities. *Nucleic Acids Research*, **44**.

4. UniProt Consortium (2019) UniProt: a worldwide hub of protein knowledge. *Nucleic Acids Res.*, **47**, D506–D515.

5. Meldal,B.H.M., Forner-Martinez,O., Costanzo,M.C., Dana,J., Demeter,J., Dumousseau,M., Dwight,S.S., Gaulton,A., Licata,L., Melidoni,A.N., *et al.* (2015) The complex portal - An encyclopaedia of macromolecular complexes. *Nucleic Acids Research*, **43**.

6. Fabregat,A., Jupe,S., Matthews,L., Sidiropoulos,K., Gillespie,M., Garapati,P., Haw,R., Jassal,B., Korninger,F., May,B., *et al.* (2018) The Reactome Pathway Knowledgebase. *Nucleic Acids Research*, 10.1093/nar/gkx1132.

7. G Csardi, T Nepusz (2006) The igraph software package for complex network research. *InterJournal, Complex Systems 1695*.

8. Aric A. Hagberg, Daniel A. Schult and Pieter J. Swart (2008) Exploring network structure, dynamics, and function using NetworkX. *Proceedings of the 7th Python in Science Conference*.

URL	Data Types	Parameters
/getData.php?	Interaction Data, tab-separated	• **id**: MANDATORY – the ID of the searched entity (UniprotKB ID for proteins, PubChem ID for chemicals or small molecules, SIGNOR-ID for SIGNOR entities)<br>• **organism**: NON-MANDATORY (default = Homo sapiens) the organism (tax ID for either Homo sapiens, Mus musculus or Rattus norvegicus) the interactions take place in
**Example: https://signor.uniroma2.it/getData.php?organism=9606&id=P62258**		
/getUniprotIDs.php	List of all UniprotKB entities curated in SIGNOR interactions (by ID)	–
/getPathwayData.php	Pathway Data, tab-separated	• **pathway**: the value identifies SIGNOR ID assigned to pathway. If used alone, it links to pathway information. In conjunction with other parameters, these will apply to the chosen pathway.<br>• **description**: does not take a value, links to pathway descriptions. If pathway perimeter is defined, shows only description for single pathway.<br>• **relations**: if no pathway is indicated, it shows all pathway relations. If a pathway is indicated and this parameter is added with no value, it will add pathway relations to result. To show only relations from a pathway, use "only" value for this parameter.

Supplementary Table 1: SIGNOR 2.0 REST API for data retrieval

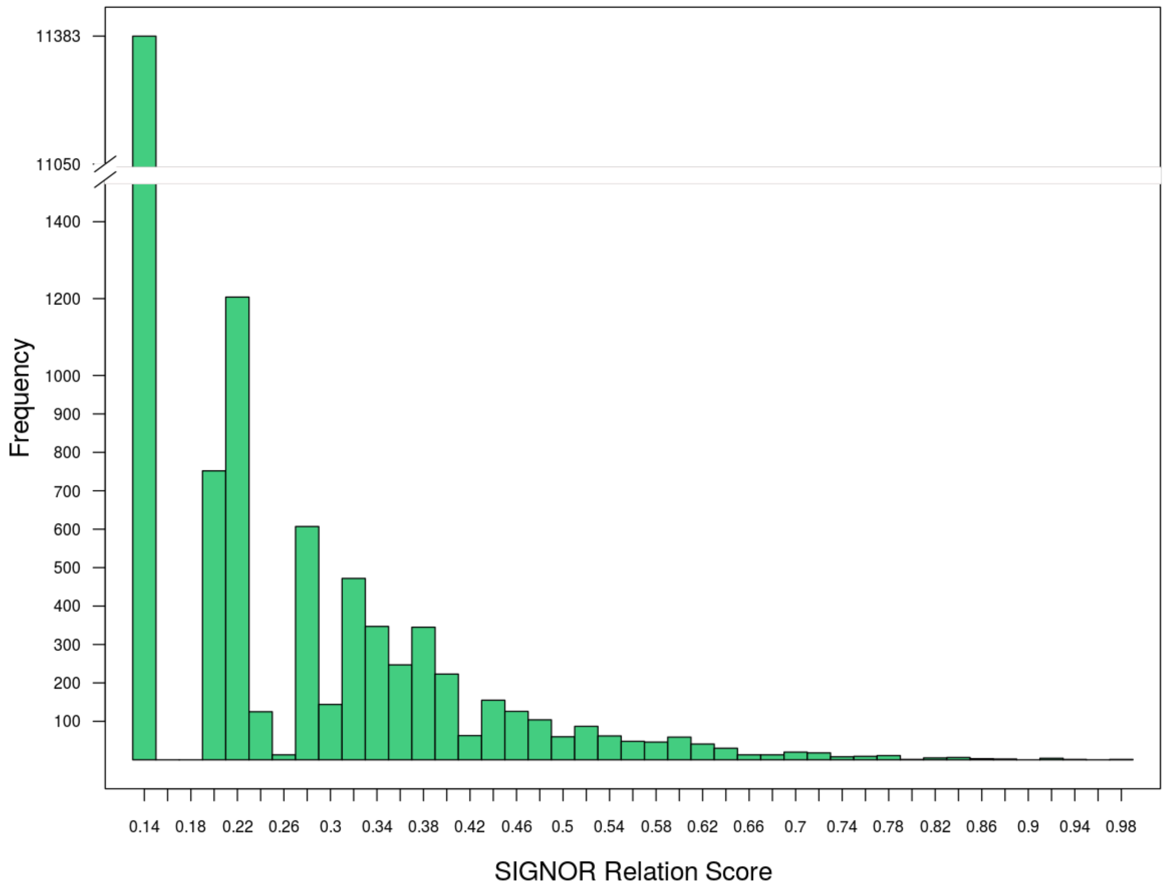

Supplementary Figure 1: SIGNOR new scoring system. The figure shows the distribution of the new SIGNOR relation score, obtained by averaging four different features (SIGNOR curated pathway occurrence, SIGNOR supporting reference occurrence, Reactome occurrences, UniProtKB protein mention).
